# Empirical research related to the ethics of pragmatic clinical trials: A scoping review

**DOI:** 10.1002/lrh2.70041

**Published:** 2025-10-04

**Authors:** Kayla R. Mehl, Stephanie R. Morain, Jeremy Sugarman

**Affiliations:** ^1^ Berman Institute of Bioethics Johns Hopkins University Baltimore Maryland USA; ^2^ Department of Health Policy & Management Johns Hopkins Bloomberg School of Public Health Baltimore Maryland USA; ^3^ School of Medicine Johns Hopkins University Baltimore Maryland USA

**Keywords:** pragmatic clinical trials, research ethics, scoping review

## Abstract

**Background:**

Pragmatic clinical trials (PCTs) offer insights into real‐world intervention effectiveness, but they may involve challenging ethical issues. Empirical ethics research may inform deliberations about them.

**Methods:**

We conducted a scoping review of empirical ethics research related to PCTs. This involved searching in PubMed and Embase, charting findings, and analyzing themes to identify trends and gaps.

**Results:**

Eighty‐two publications were included, which examined a vast number of issues encompassing 22 themes. The five most prominent were: consent/disclosure; risk assessment; trust and transparency; burdens, barriers, and costs; and engagement. Written consent is often impractical, prompting interest in opt‐out or general notification approaches. Challenges in risk assessment include variability in defining minimal risk, thereby complicating regulatory determinations for the appropriateness of particular participant protections and communicating research risks. Trust‐building practices, such as result‐sharing and data‐use disclosure, can foster confidence. Stakeholder engagement can address logistical barriers, improve recruitment, and align research with participant needs. Time, financial, and regulatory burdens are significant obstacles to implementing PCTs.

**Conclusion:**

There has been progress in understanding many ethical issues encountered in PCTs, including appropriately navigating alternatives to obtaining written informed consent, trust‐building, and the operational role of stakeholder engagement. However, critical gaps remain, with research concentrated in Western contexts and reliant on surveys and hypothetical scenarios, limiting generalizability and real‐world insights. Addressing these gaps with geographically inclusive studies, innovative methods, and nested empirical work will be important for more comprehensively understanding the ethical issues in PCTs and developing appropriate approaches to mitigating them.

## 1 BACKGROUND

Pragmatic clinical trials (PCTs) are designed to evaluate the effectiveness of interventions in real‐world conditions, often by embedding research within routine clinical care. The distinction between pragmatic and explanatory trials was first articulated by Schwartz and Lellouch in 1967, who described two distinct “attitudes” toward trial design.^1^ The explanatory attitude seeks to test hypotheses about biological mechanisms under tightly controlled conditions, whereas the pragmatic attitude aims to inform medical decision‐making in the circumstances of usual practice. Subsequently, this distinction has been summarized in shorthand as a contrast between trials conducted under ‘ideal’ conditions and those conducted under ‘real‐world’ conditions (e.g., U.S. Congress Office of Technology Assessment, 1978).^2^ More recently, it has been recognized that trials rarely fall entirely into one category or the other; rather, pragmatism is best understood as a continuum, as reflected in the PRECIS‐2 framework.^3^

Because PCTs are often embedded in routine clinical care and evaluate interventions already in use, they give rise to ethical questions that differ from those posed by traditional explanatory trials. These include questions about whether consent should more closely resemble the sometimes truncated practices used in clinical care or those typically elaborated in explanatory research,^4^, ^5^, ^6^ how to define ‘minimal risk,’^7^, ^8^ and what role data monitoring and oversight should play.^9^, ^10^

While much ethics scholarship on PCTs has been conceptual,^9^, ^11^, ^12^ there is a growing body of empirical research—that is, research that uses the tools of the social sciences to examine how ethical issues in PCTs are experienced, interpreted, and addressed by stakeholders such as patients, clinicians, investigators, oversight bodies, and healthcare leaders. Empirical ethics can complement conceptual analysis by grounding normative arguments in lived experience and revealing which issues are ethically salient in practice. In doing so, it may also illuminate where ethical aims are challenged or reshaped by the realities of clinical research practice.

A number of reviews have explored specific ethical domains or presented normative analyses related to PCTs and learning health systems,^11^, ^13^, ^14^, ^15^ however, none have provided a comprehensive synthesis of the full range of empirical ethics research related to PCTs. This scoping review aims to fill that gap by broadly mapping the empirical literature. Our goal is to bring greater visibility to the diversity of ethically relevant concerns that have been explored empirically, highlight where those efforts have been concentrated, and identify areas where further inquiry is needed. In doing so, this review provides a foundation for more in‐depth analysis of the descriptive patterns, thematic gaps, and normative implications of existing empirical ethics research on PCTs.

## 2 METHODS

A scoping review of the empirical ethics literature related to PCTs was conducted in line with recommended best practices, and involved five steps, as detailed below.^16^, ^17^ No review protocol was developed or registered for this scoping review.

### 2.1 Identifying the Research Question

Our guiding research question was, “What empirical research has been conducted on ethically relevant topics in PCTs?” While this question spans a broad array of related approaches and terms—including comparative effectiveness research (CER), research on medical practice (ROMP), learning health systems (LHS), low‐risk randomized controlled trials (lrRCTs), cluster randomized trials (CRT), and point of care research (POC‐R)—we curated our review to incorporate only empirical work on ethics‐related concerns that are directly relevant to PCTs.

Given the conceptual overlap between PCTs and these related domains, we adopted an inclusive approach that prioritized publications that either explicitly described a trial as pragmatic or addressed interventional research designs consistent with pragmatic aims. While some publications included in our review describe trials embedded in real‐world clinical settings, we did not limit inclusion to that criterion, nor did we apply formal measures of pragmatism such as the PRECIS‐2 tool. While we aimed to capture the range of empirical ethics work relevant to interventional research typically associated with pragmatic goals, we excluded publications that focused solely on observational research (e.g., some work on LHSs).

### 2.2 Identifying Relevant Publications

We identified relevant publications via PubMed and Embase searches conducted between February and April 2024. The search strategy was developed collaboratively with an informationist, though it was not formally peer‐reviewed using the PRESS guideline.^18^ In PubMed, we used Boolean logic operators (e.g., AND, OR, NOT), Medical Subject Headings (MeSH) terms, and title/abstract (tiab) searches (Online Appendix A). In Embase, we used Boolean logic operators, main topic searches (topic), and searches within abstracts, titles, and keywords (ab:ti:kw) (Online Appendix B). We included related terms such as CER, ROMP, pragmatic CRTs, and LHSs.

We then performed both backward and forward citation tracking using reference lists and Google Scholar's “Cited by” function.

### 2.3 Study Selection

Eligible publications were uploaded to Covidence,^19^ a data screening and extraction software program. Two reviewers (KRM, SRM) independently screened the titles and abstracts of identified publications using Covidence's built‐in Yes/No/Maybe tool to determine which would advance to full‐text screening. Discrepancies were discussed, and when consensus could not be reached or the decision was “Maybe,” the publication was advanced to full‐text screening. At the full‐text stage, both KRM and SRM assessed all publications using Covidence's Include/Exclude tool. Discrepancies were resolved through discussion; if consensus could not be reached, SRM made the final inclusion/exclusion decision.

The following inclusion and exclusion criteria were used to determine study eligibility:

Inclusion:

- Peer‐reviewed journal articles, systematic reviews, and book chapters.
- Empirical publications (qualitative, quantitative, or mixed methods) that present original data or synthesize empirical findings.
- Publications that centrally examine ethical issues related to the design, conduct, or oversight of interventional research commonly associated with pragmatic trial design (e.g., PCTs, CER, ROMP, pragmatic CRTs, LHSs).
- Publications in English.

Exclusion:

- Gray literature (e.g., conference abstracts, theses).
- Publications focused exclusively on explanatory trials, observational studies, or other trial types that do not address ethical issues relevant to PCTs.
  Publications that do not present or systematically synthesize empirical findings.
- Publications that do not present or synthesize empirical findings.
- Publications that do not substantively engage with ethical issues.

### 2.4 Charting the Data

Data were extracted using a Covidence template designed by KRM with SRM's input. Extracted variables included: publication characteristics (title, author[s], publication year, country/region); study aim; terminology used (e.g., PCT, CER, CRT); participant group; study method; and ethical themes, results, and additional notes (e.g., notable details or quotes). The initial version of the template was informed by notes KRM took during full‐text review on study methods, ethical topics, terminology, participant groups, and country of origin. The terminology used to describe participant groups varied across publications, so we categorized them as accurately as possible based on the information provided (Table 1).

**TABLE 1:** Definitions and distribution of participant groups.

Participant groups	Count
Researchers *Individuals involved in designing, conducting, analyzing, and refining PCTs. This group includes principal investigators, clinical researchers, study staff, and methodologists who focus on statistical design, randomization, and other methodological aspects of trials*.	25
Public/Citizens *Members of the general public who participate in surveys, interviews, or other engagement activities, providing insights on community attitudes, ethical considerations, or patient preferences*.	23
Patients *Individuals receiving healthcare services who are actual or potential participants in PCTs. This group represents the primary target population of clinical interventions being tested*.	22
Healthcare Professionals (HCPs) *Medical professionals such as doctors, nurses, and allied health practitioners who provide patient care and may play roles in implementing PCTs within healthcare settings*.	19
IRB/RECs *Members of Institutional Review Boards (IRBs) or Research Ethics Committees (RECs) who review and approve PCT protocols to ensure they meet ethical and regulatory standards*.	13
Caregivers/Parents *Family members or informal caregivers who provide care to patients, usually those who are more vulnerable like children, older adults, and/or ill or disabled individuals*.	7
Healthcare Leaders *Individuals in administrative or strategic roles within healthcare systems, such as hospital directors, clinical managers, or healthcare executives, who influence how PCTs are conducted or integrated into routine care*.	7
Studies *This category serves as a placeholder for reviews that analyze trial data or methodologies rather than featuring specific participant groups*.	6
Patient Partners *Patients who take on active advisory or co‐design roles in PCTs, contributing their lived experience to shape trial design, conduct, and analysis*.	6
Financial Stakeholders *Includes both funders—organizations that provide financial support for PCTs* (e.g., *NIH, PCORI*)*—and payers—organizations that assess the cost‐effectiveness of interventions and manage the financial reimbursements for them (e.g., insurance companies, Medicare and Medicaid, National Health Service, Provincial Health Ministries)*.	5
Regulatory and Policy Authorities *Entities responsible for creating, setting, and enforcing policies and regulations that govern the conduct of PCTs. These participants develop legislative frameworks, ensure compliance with legal requirements, and uphold safety and ethical standards in healthcare research (e.g., FDA, EMA)*.	4
Ethicists *Specialists in ethics who offer guidance on complex moral issues in PCTs, including consent, risk, equitable inclusion, and other ethical principles*.	3
Lawyers *Legal professionals who advise on regulatory compliance, patient rights, trial contracts, and other legal aspects of PCTs, ensuring adherence to applicable laws and regulations*.	2
Community Advisory Group *Groups of community representatives who provide feedback and guidance on PCTs to ensure that trial design, implementation, and outcomes are relevant to and respectful of community needs*.	1

Midway through data extraction, SRM reviewed a 10% sample and provided feedback. KRM revised the Covidence template and previously extracted data, then completed the remaining extractions using the updated template.

To identify ethical themes, we used an inductive, interpretive approach based on how study authors framed ethical concerns. Rather than applying a pre‐established set of ethical categories (e.g., autonomy, justice), we derived themes from the language and conceptual framing used in the included publications. During data extraction, KRM copied relevant excerpts of text (e.g., descriptions of study aims, findings, or ethical commentary) into a working document, which supported the development of a shared lexicon for identifying and defining key ethical themes.

A theme was coded as present in a given publication only if it was discussed with sufficient depth and substance to be considered a central ethical focus. The themes were applied consistently using the evolving lexicon, and any ambiguities about how to categorize or interpret themes were resolved collaboratively by KRM and SRM.

Although some themes are conceptually interrelated and often overlapped (e.g., consent/disclosure and autonomy), we coded them as distinct. This preserved analytic clarity and flexibility, allowing us to capture the diversity of how ethical issues were framed and emphasized across publications.

### 2.5 Collating, Summarizing, and Reporting Results

KRM developed a manual color‐coding system to sort excerpts of ethically salient text into preliminary ethical themes. These themes were iteratively refined through close reading and comparison. To support consistency, an evolving lexicon of definitions was created and used throughout the thematic coding process. A third pass through the working document of coded excerpts was conducted to ensure internal coherence and finalize the thematic categories. No qualitative software or formal coding framework was used, but the process was systematic, inductive, and reflective.

The final results were organized thematically using a narrative format, consistent with guidance for scoping reviews provided by Arksey and O'Malley. The five prominent themes emphasized in the thematic analysis were selected based on their prevalence across the full set of included publications.^16^ To complement the thematic analysis, we used Excel^20^ and Tableau,^21^ a data visualization software, to analyze data and create visualizations of publication trends by region, year, terminology, and methods.

## 3 RESULTS

### 3.1 Study Selection

**FIGURE 1:**
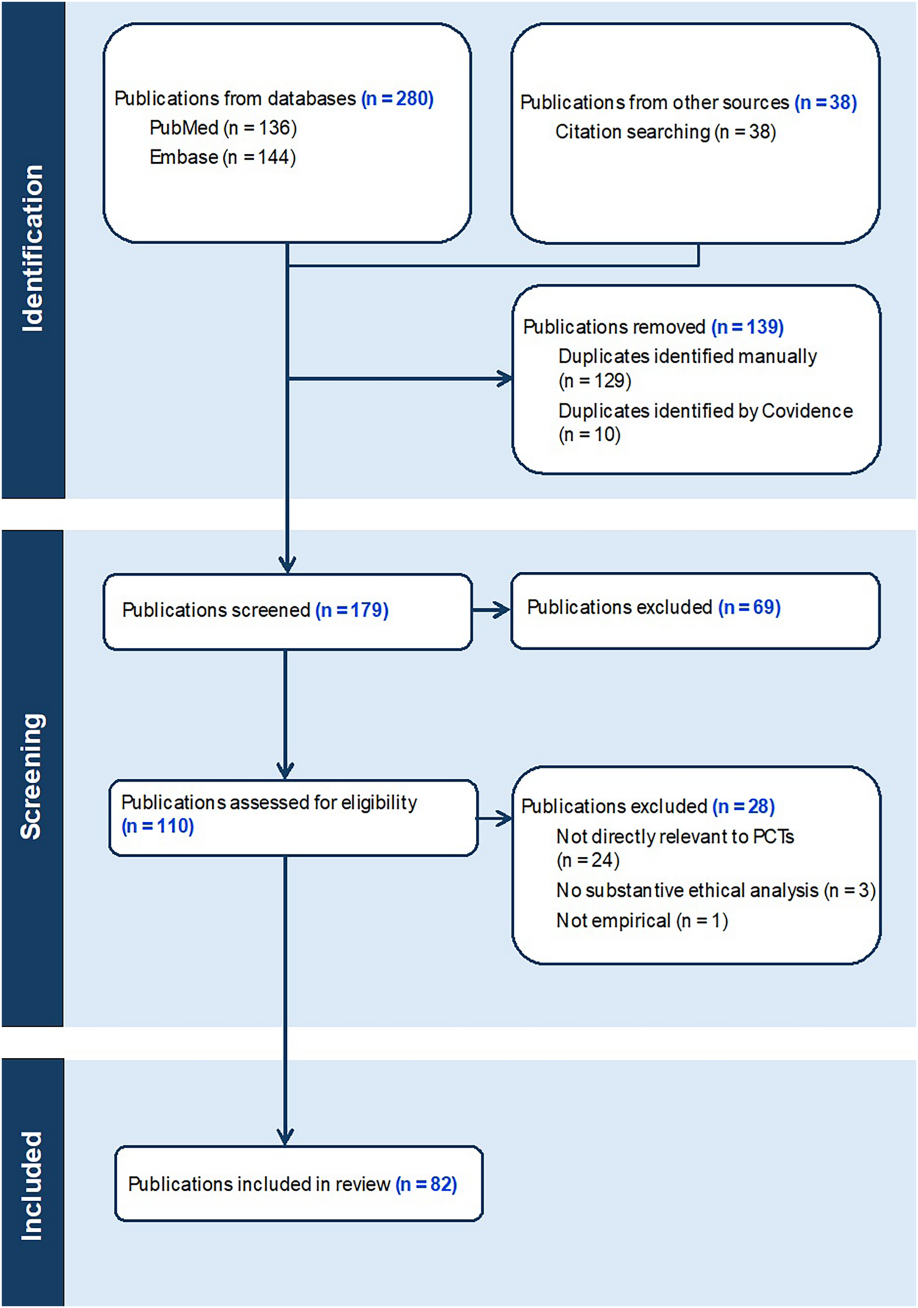
PRISMA flowchart of publications selection.

The database search yielded 179 publications. After title and abstract screening, 110 publications were selected for full‐text review, and 69 were excluded. Of the 110 full‐text publications, 28 were excluded: 24 were irrelevant, 3 lacked empirical data, and 1 was non‐empirical. Ultimately, 82 publications met the inclusion criteria and were compiled for data extraction (see Online Appendix C). The PRISMA flow diagram illustrating the study selection process is presented in Figure 1.

### 3.2 Study Descriptors

Empirical publications on PCT ethics have steadily increased from 2010 to 2024.

Most of the data in this review derive from the United States (*n* = 64), followed by Canada (*n* = 13), the UK (*n* = 11), Australia (*n* = 9), and New Zealand (*n* = 6). Some publications presented data from multiple countries/regions (*n* = 11); these multi‐country studies were counted separately for each included country, resulting in country‐specific totals exceeding the overall number of included studies (*n* = 82).

### 3.3 Methods

Surveys (*n* = 39) were the most common method, followed by key stakeholder interviews (*n* = 21), mixed methods (*n* = 7), focus groups (*n* = 6), systematic/scoping reviews (*n* = 6), and deliberative methods (*n* = 3) (Table 2). Of the 39 publications that used quantitative survey methods alone (i.e., not as part of a mixed‐methods design), 20 involved descriptive surveys, while 19 were experimental surveys. One of the experimental survey publications used a discrete choice experiment (DCE), a method that presents participants with structured trade‐offs to quantify preferences among competing trial attributes.^22^ Most publications used hypothetical scenarios (*n* = 45), while others gathered general perspectives on PCTs without being tied to specific trials (*n* = 21), were nested in ongoing PCTs (*n* = 7), or used real‐world examples of PCTs (*n* = 3). The remaining 6 publications were systematic or scoping reviews synthesizing empirical findings rather than presenting original data.

**TABLE 2:** Distribution of study methods.

Study method	Count
Survey *Quantitative research using structured questionnaires to collect data on attitudes, experiences, or knowledge from a defined group*.	39
Key stakeholder interview *Qualitative studies analyzing perspectives of those directly affected by a study or intervention, including unsolicited communications when systematically examined*.	21
Mixed methods *Studies that combine two or more distinct research methods—qualitative, quantitative, or both—to explore a research question*.	7
Focus group *Guided group discussions used to explore participants' views, experiences, or reactions to a topic, typically involving interaction among participants*.	6
Review *A synthesis of existing literature, either systematically or narratively, to summarize and assess the current state of knowledge on a topic*.	6
Deliberative methods *Structured discussions that engage stakeholders in reflective dialogue about complex issues, often to inform policy or ethical decision‐making*.	3

### 3.4 Participants

**FIGURE 2:**
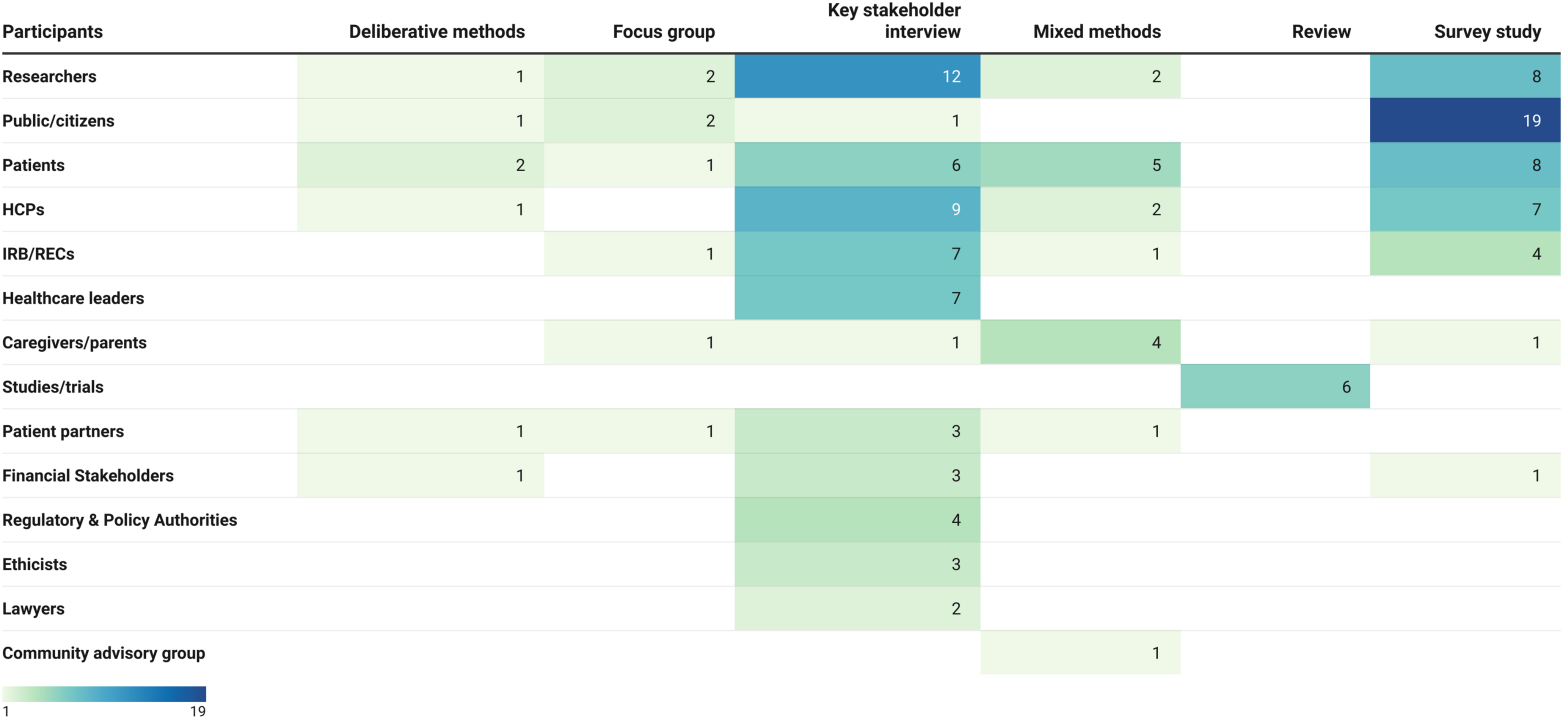
Distribution of study method by participant group.

Researchers (25/82) were the most studied group, followed by the public (23/82), patients (22/82), healthcare professionals (HCPs) (19/82), and institutional review boards or research ethics committees (IRB/RECs) (13/82) (Table 1). Researchers and HCPs primarily participated in interviews, addressing consent, engagement, and burdens. Surveys were primarily used in publications involving public and patient participants, which tended to focus on consent, trust, and willingness to participate. Publications involving IRB/RECs primarily addressed issues related to consent and risk (Figure 2).

### 3.5 Ethical Themes

We identified 22 ethical themes discussed in the included publications (Table 3). These distributions offer a high‐level view of the topics that appeared most frequently across all publications included in the review. The most prevalent were: (1) Consent/disclosure (49/82), (2) Risk assessment (23/82), (3) Trust and transparency (18/82), (4) Burdens/barriers/costs (18/82), and (5) Engagement (17/82).

**TABLE 3:** Definitions and distribution of main themes.

Main themes	Count
Consent/Disclosure *Refers to how participants are informed about their involvement in PCTs, including traditional consent and alternative approaches such as general notification, opt‐in, opt‐out, verbal, deferred, or waived consent. This theme addresses ethical concerns related to autonomy, transparency, voluntariness, and participant understanding in the context of routine care*.	49
Risk Assessment *Refers to the assessment of harms or negative outcomes (both actual and perceived) arising from the trial by various stakeholders*.	23
Trust and Transparency *Refers to the need for clear, honest communication that fosters participant confidence in the research, ensuring they feel informed and respected. It emphasizes the importance of providing adequate, accurate information to build trust and maintain ethical integrity*.	18
Burdens, Barriers, and Costs *Refers to the ethical and practical burdens that all stakeholders (*e.g., *participants, researchers, healthcare institutions or healthcare providers) may face, such as time commitments, logistical or regulatory challenges, or financial costs*.	18
Engagement *Refers to the involvement of stakeholders—such as patients, healthcare providers, and community members—in trial design, implementation, and evaluation, often focusing on how this involvement influences the acceptability, feasibility, rigor, and relevance of the trial. It also encompasses the engagement of study participants through processes like informed consent, disclosure, and result‐sharing*	17
Duty or Willingness to Participate *Involves the motivations and barriers that influence participants' decisions to join or remain in a trial. It covers factors such as altruism, social responsibility, perceived benefits, mistrust, or perceived risks, along with the balance between duty and voluntary participation*.	16
Patient Understanding *Refers to whether participants have a clear understanding of the trial's purpose and methodology, and how this influences other PCT‐related factors, including their willingness to participate or consent/disclosure preferences*.	11
Social Value *Encompasses the broader societal benefits that a trial aims to generate, such as public health improvements or healthcare delivery enhancements*.	10
Data Sharing and Management *Refers to how participant data is collected, stored, shared, and protected in PCTs*.	10
Autonomy *Involves the limitations on decision‐making for both physicians and patients—such as restrictions on informed consent or intervention choice—which may impact their willingness to participate*.	9
Oversight and Regulatory Challenges *Examines the role of regulatory and ethical review bodies, such as IRBs and RECs, in monitoring PCTs, including their intended responsibilities, current challenges or shortcomings, and potential areas for improvement*.	9
Justice/Equity *Assesses fair access and equitable distribution of burdens and benefits, focusing on the inclusion of diverse populations—especially marginalized or vulnerable groups—and the reduction of healthcare disparities*.	8
Cluster Randomization *Addresses the ethical implications of assigning groups (*e.g., *clinics, hospitals) to different interventions rather than individual randomization*.	8
Research‐QI‐Clinical Practice Distinction *Refers to the ethical and regulatory challenge of differentiating between clinical research, quality improvement, and clinical practice, often referred to as the “gray zone.”*	8
Equipoise *Refers to the ethical justification for conducting a trial, ensuring that there is genuine uncertainty among clinicians about which intervention is better*.	7
Support for Embedded Research *Refers to participants' approval of integrating PCTs into healthcare settings, reflecting support for the concept rather than a personal willingness to participate*.	7
Usual, Routine, or Individualized Care *Assesses the ethical implications of usual care, including, for example, whether it meets individual patient needs and adheres to ethical standards*.	6
Randomization *Refers to the ethical implications of randomizing participants to different interventions, including the justification and its effects on treatment and care*.	6
Research Quality *Involves assessments of whether PCTs maintain high standards of scientific rigor, validity, or reliability*.	5
Implementation and Sustainability *Explores the ethical and logistical challenges of implementing PCTs in real‐world settings, along with their feasibility and long‐term sustainability*.	4
Collateral Findings *Involves the unexpected findings (*e.g., *incidental medical results) that may be discovered during the trial are managed*.	3
Returning Aggregate Results *Examines the ethical considerations of sharing aggregate trial results with participants, including trials conducted under a waiver of consent*.	2

### 3.6 Thematic Analysis

We conducted a narrative synthesis of the five most prevalent ethical themes, as described below:

#### 3.6.1 Consent/Disclosure

Consent/disclosure has been examined not only in terms of traditional written consent but also alternative approaches to disclosure and authorization such as general notification, opt‐in, opt‐out, oral, deferred, and waived consent. Transparent communication and participant choice are widely valued, though acceptable approaches vary by context and perceived risk. Simplified approaches are considered appropriate in low‐risk or system‐level studies, especially when paired with opt‐out options—but passive or notification‐only strategies raise concerns about reduced understanding, compromised voluntariness, and diminished trust, particularly in trials affecting individual treatment decisions.

##### 3.6.1.1 Stakeholder Preferences

Several publications report that patients and physicians value transparency and active engagement, especially in trials affecting individual treatment.^23^, ^24^ Some publications also found support for simplified consent methods including brief oral consent or general notification—when accompanied by respect‐enhancing practices, such as ongoing information sharing and access to study results.^25^, ^26^, ^27^ In low‐risk settings, simplified consent approaches tend to be viewed as acceptable by patients and IRB/RECs, provided they are accompanied by an explicit and accessible opt‐out option.^28^, ^29^, ^30^, ^31^, ^32^

However, in a few publications various stakeholders—including patients, IRB members, and patient advocates—raised concerns that streamlined approaches, particularly notification‐only approaches and complete waivers of consent, may inadequately inform potential participants, reduce their awareness of enrollment, or undermine voluntariness.^33^, ^34^, ^35^, ^36^

##### 3.6.1.2 Context‐Specific Preferences and Variability

Consent preferences varied across publications and appeared to be influenced by clinical context. For example, Dickert et al. found strong support (73%–80%) for prospective consent in trials testing approved medications and procedural interventions in acute care settings.^37^ By contrast, other publications report that general notification was considered sufficient by HCPs and members of the general public for some operational studies, such as those evaluating hand‐washing protocols.^23^, ^24^ In nephrology, one publication found that patients expressed similarly high acceptance of opt‐in (84%), opt‐out (82%), and notification‐only (81%) approaches, with less support for no‐notification (66%).^22^ Yet in another nephrology‐focused study, patients with end‐stage renal disease and their nephrologists preferred opt‐out notification over formal written consent, citing concerns about patient burden and information overload.^28^

##### 3.6.1.3 Consent, Trust, and Transparency

The role of trust in shaping consent preferences, though in varying ways, was mentioned across publications. For example, a few publications report that patients preferred to learn about PCTs through their regular physicians, whom they viewed as trustworthy sources suited to explaining trial implications and uncertainties.^32^, ^38^, ^39^, ^40^ In other cases, patients' trust appeared to hinge less on who delivered the information and more on whether the consent process was transparent, understandable, and respectful of their autonomy.^23^, ^40^, ^41^

Consent approaches can also shape perceptions of transparency and voluntariness.^29^, ^33^, ^37^ In a national survey experiment using hypothetical research scenarios, Weinfurt et al. found that active approaches to consent—such as oral and written consent—were viewed as more acceptable (77%–93%) than passive approaches such as general notification (31%–57%). Despite the inclusion of an opt‐out option, 21%–36% of participants assigned to general notification were unaware they would be enrolled by default, raising concerns about compromised voluntariness.^33^ Another publication further emphasized the importance of explicit disclosure: for instance, study‐specific disclosures with explicit opt‐out options were viewed by some patients and IRB members as a more acceptable balance between operational efficiency and ethical commitments to transparency and autonomy—particularly when compared to broader institutional notifications or study‐specific disclosures that lacked an explicit opt‐out.^29^

#### 3.6.2 Risk Assessment

Stakeholder assessments of risk in PCTs may vary not only by role and setting, but also by how risks were defined—clinical, informational, institutional, or relational. While some view PCTs as low‐risk due to their use of routine care, others emphasize that minimal risk is context‐dependent and shaped by population vulnerability, data practices, and governance structures. Misconceptions and misunderstandings—especially regarding intervention novelty or enrollment procedures—frequently influence perceptions, even when information is accurately conveyed. Across publications, risk is understood not as a fixed feature of the intervention, but as emerging from its social and institutional environment. We describe relevant factors in turn.

##### 3.6.2.1 Differences by Stakeholder Role

Perceptions of risk often track with professional responsibilities. For instance, IRB members and ethics professionals tend to focus on regulatory obligations and exercise caution when assessing cluster designs, vulnerable populations, or comparisons of standard‐of‐care interventions.^14^, ^42^, ^43^, ^44^ Some researchers, by contrast, emphasize the routine nature of tested interventions and minimize the need for additional safeguards.^14^, ^29^ Patients and advocates, meanwhile, often emphasize relational and informational risks, such as loss of transparency or control over data, over direct clinical harms (although some also raise safety concerns about standard treatments, likely due to confusion about the study design).^23^, ^40^ Healthcare leaders focus on institutional and reputational risks, especially in multicenter trials and data‐sharing contexts.^45^

##### 3.6.2.2 Importance of Context

Stakeholders tend to view risk not as inherent to the intervention but shaped by contextual factors such as the care environment, population vulnerability, and strength of oversight mechanisms. Concerns are heightened in trials conducted in under‐resourced or heterogeneous care settings, where the label of “minimal risk” may be seen as potentially misleading.^14^, ^44^ Risk assessments also shift in relation to data practices. In settings with weak data governance or potential for social harm, the reuse of EHR data without consent may raise concerns about discrimination or loss of trust.^26^, ^46^

##### 3.6.2.3 Misconceptions and Communication Challenges

Several publications document misconceptions that may influence perceived risk, particularly the belief that PCTs involve unproven or unsafe interventions. Patients in some publications expressed disinterest toward research participation due to fears that standard treatments were experimental or riskier than they were.^23^, ^47^ Simplified consent approaches, especially notification‐only, sometimes created confusion about enrollment and oversight, which shaped both understanding and comfort with participation.^33^, ^41^ In emotionally fraught contexts like neonatal care, these communication issues amplified concerns.^48^

##### 3.6.2.4 Informational Risks and Data Use

Informational risks—including reidentification, unauthorized data use, or reputational harm—were central to risk assessments, especially when consent was waived or streamlined. These concerns are salient across roles and especially acute in settings where public trust was fragile.^14^, ^45^, ^46^ Even when data were de‐identified, stakeholders emphasized that opaque data governance frameworks heightened perceptions of risk.^26^, ^44^ As a result, some patients expressed a preference for traditional consent to maintain a sense of control.^39^, ^49^

#### 3.6.3 Trust and Transparency

Across publications, trust and transparency in PCTs were shaped by how clearly information was communicated, who delivered it, and how research activities were governed. Misunderstandings about PCTs were common, and trust in health care professionals often influenced comfort with participation. However, trust could be undermined by vague institutional motives, passive communication, or weak oversight. Stakeholders across roles and settings emphasized that ethical transparency requires not just disclosure, but meaningful communication, accountable governance, and alignment with patient and community interests.

##### 3.6.3.1 Communication, Understanding, and the Boundaries of Transparency

Across publications, stakeholders emphasize that trust in PCTs depends not only on whether information is disclosed, but also on how clearly, accessibly, and meaningfully it is communicated. Patients and members of the public frequently misunderstand the nature of PCTs, often assuming they involve experimental treatments or placebos rather than comparisons of routine care—what some describe as an “investigational misconception.”^23^, ^47^ While transparency is generally valued, several publications highlight concerns that overly technical or poorly targeted communication—such as passive notifications or jargon‐heavy disclosures—could undermine, rather than enhance, trust.^35^, ^46^ IRB members and health system leaders express uncertainty about what constitutes adequate or ethically appropriate transparency, particularly when disclosing study details might confuse patients or affect their perceptions of care.^42^, ^50^ Together, these findings suggest that transparency is not ethically meaningful unless paired with communication strategies that promote genuine understanding across diverse patient populations.

##### 3.6.3.2 Trust in Institutions and HCPs

Across several publications, perceptions of trust in health care institutions and HCPs shape how stakeholders evaluate the ethical acceptability of PCTs. Patients consistently report greater comfort receiving research‐related information from their own physicians, with 72.6% preferring this for randomized studies and 67.0% for studies using medical record review.^40^ Clinicians are often viewed as trusted advocates, and disclosure by a familiar HCP enhanced trust in the research process.^35^, ^40^ However, trust in HCPs does not uniformly translate into support for streamlined consent; some participants prefer fuller disclosure when institutional motives are unclear or data use appears to be commercially driven.^29^, ^50^ In Kenyan health settings, where data involve stigmatized conditions such as HIV, transparency and community input are seen as vital for maintaining public trust.^46^ In high‐income settings, some stakeholders caution that waiving consent—particularly without clear communication about risk, oversight, or the rationale for streamlined disclosure—may jeopardize public trust in pragmatic trials.^35^ These findings underscore that both interpersonal and institutional trust depend on perceived alignment with patient interests and clear, ethical governance.

##### 3.6.3.3 Oversight, Accountability, and Ethical Governance

Several publications highlight concerns about the adequacy and appropriateness of ethical oversight in PCTs, especially when such research is embedded within clinical care. IRB members report uncertainty about how to assess risk and distinguish research from quality improvement, particularly in trials comparing standard‐of‐care interventions.^42^ Health system leaders describe existing IRB processes as misaligned with learning health system goals, noting that current oversight models often delayed or discouraged low‐risk research.^50^ In Kenya, stakeholders emphasized the need for stronger, context‐specific governance to ensure local accountability—especially when data reuse involved external researchers or stigmatized conditions.^46^ In the U.S. context, patients also express discomfort with being included in research without their knowledge, even when risks were low, underscoring the importance of visible and trustworthy oversight mechanisms.^23^ Across publications, stakeholders call for clearer, proportionate, and more transparent governance frameworks that both protect participants and support ethically responsible learning within health systems.^40^

#### 3.6.4 Burdens, Barriers, and Costs

Burdens, barriers, and costs in PCTs were examined in multiple publications in relation to operational feasibility, professional role strain, consent processes, and data governance. Different stakeholders (including clinicians, researchers, and institutional leaders) described a range of logistical, ethical, and infrastructural challenges that contributed to trial delays, increased workload, and resource demands. While some burdens stem from trial implementation within routine care, others arise from regulatory expectations and unclear guidance, particularly around consent and data sharing. Together, these findings illustrate how practical and ethical complexities can undermine the feasibility and equitable execution of PCTs.

##### 3.6.4.1 Operational and Structural Barriers

Operational and structural burdens are a recurring concern across multiple publications. Physicians and healthcare staff emphasize that participation in PCTs often created additional time and workflow burdens, particularly when research activities disrupt routine clinical operations or are perceived as poorly integrated into existing systems.^24^, ^51^ Some stakeholders express concern that these burdens could negatively affect clinician‐patient relationships or compromise care quality if not adequately managed.^50^, ^51^ Research in under‐resourced settings, including Aboriginal Medical Services in Australia, highlight the challenges posed by workforce shortages, lack of infrastructure, and funding models that disincentivize trial participation.^52^ Others point to logistical barriers such as IRB coordination, data system fragmentation, and the cultural shifts required to build and sustain embedded research infrastructures.^53^, ^54^ Across publications, these burdens are seen as potentially undermining the feasibility, sustainability, and ethical implementation of PCTs.

##### 3.6.4.2 Professional Role Conflicts and Consent‐Related Challenges

Some publications identified professional role conflicts as a source of burden in PCTs. In qualitative interviews, physicians described discomfort with being expected to facilitate recruitment or deliver study‐related information without having been consulted during trial design, particularly when protocols disrupted clinical workflow or introduced documentation requirements.^24^ Veterans Affairs HCPs similarly emphasized tensions between clinical autonomy and protocol compliance, raising concerns about role confusion and negative impacts on the provider–patient relationship.^51^ In for‐profit settings, stakeholders noted ethical friction between research goals and institutional or business responsibilities, complicating expectations around clinician gatekeeping.^55^ Health system leaders also described broader ambiguity around the clinical–research boundary and noted that unclear or shifting responsibilities created operational strain.^50^

Other publications examined how consent processes and related decision‐making about ethical oversight contribute to stakeholder burden. In a discrete choice experiment, physicians were less willing to participate in trials using notification‐only or no‐notification models, suggesting that streamlined approaches may conflict with professional norms and impose ethical or emotional discomfort.^22^ Investigators also reported delays, costs, and recruitment barriers stemming from inconsistent ethics review processes and lack of clear consent guidelines in cluster randomized trials.^56^

##### 3.6.4.3 Data Governance and Ethical Concerns about Sharing

Several publications identified data governance and sharing as sources of ethical and operational burden in PCTs. In interviews with stakeholders (including investigators, data stewards, and regulatory officials), respondents described the administrative demands of negotiating data use agreements, managing regulatory compliance, and implementing security protections as time‐ and resource‐intensive.^26^, ^45^ These burdens were viewed as especially complex when data were collected under a waiver or alteration of consent, which some respondents felt heightened institutional responsibility and raised concerns about participant autonomy.^26^ Organizational stakeholders (including healthcare system leaders, researchers, and IRB/REC members) also cited opportunity costs and staffing constraints associated with preparing data for privacy‐preserving methods, particularly when those methods limited analytic flexibility.^45^ Other publications highlighted the logistical and technical challenges of building governance infrastructure and harmonizing data systems across research sites.^53^, ^54^ Together, these findings suggest that ethical and regulatory safeguards may introduce practical barriers that complicate the conduct and scalability of PCTs.

#### 3.6.5 Engagement

Across publications, engagement in PCTs is framed primarily in instrumental terms and implemented in ways that vary widely in scope, timing, and depth. While many researchers and stakeholders emphasize engagement's potential to enhance the relevance, feasibility, and implementation of trials, fewer describe it as ethically necessary. Instead, engagement practices are often limited, inconsistently applied, and shaped by structural and relational barriers. Together, these publications highlight a persistent gap between the ideal of inclusive, sustained collaboration and the realities of constrained resources, power dynamics, and unclear stakeholder roles.

##### 3.6.5.1 Emphasis on the Instrumental

Stakeholder engagement in pragmatic trials is most often described as a way to enhance the relevance, feasibility, and uptake of research rather than as a means of fulfilling ethical obligations, such as respect, inclusion, or justice. Researchers and patients involved in PCORI‐funded trials emphasized the value of engagement for refining study questions, aligning outcomes with stakeholder priorities, and boosting implementation, but rarely framed it as ethically required.^57^ Investigators from PCORI Pilot Projects likewise highlighted the instrumental benefits of engagement, with few describing shared leadership or citing ethical justifications.^58^ Health researchers responding to a national survey echoed these patterns, prioritizing engagement for its strategic value rather than its moral significance.^59^ Although equity‐related concerns were occasionally raised—such as in a study of hemodialysis trials—operational goals remained the dominant focus.^55^ Survey data from Vanderhout et al. further confirmed that research quality and applicability—not ethical commitments—were the dominant rationales for involving patients or the public.^60^

##### 3.6.5.2 Variation in Scope, Timing, and Depth of Engagement

Across publications—many of them based on analyses of PCORI‐funded trials—engagement practices in PCTs varied widely in scope, timing, and depth. In a review of 126 peer‐reviewed publications from PCORI‐funded projects, only 37% described engagement consistent with shared leadership or collaboration, while 46% relied on consultative approaches and 12% described one‐way input only.^61^ Survey data from PCORI's Pilot Projects similarly showed that although 90% of investigators engaged patients, engagement was often limited to early stages such as topic selection or protocol development, with fewer involving patients in data analysis or dissemination.^58^ Qualitative interviews with patients and investigators reinforced this pattern: while some patients reviewed study materials, few were engaged in shaping research questions or design decisions, and their input had limited impact on study direction.^57^ Survey data from a broader sample of health researchers similarly found that while many had experience involving patients or caregivers, engagement most often occurred during topic selection, recruitment, or dissemination, with far fewer involving patients in data analysis (12%) or comparator selection (28%).^59^ However, determining how consistently these practices are implemented remains difficult: a recent international survey found that although 47% of pragmatic trialists reported involving patients or the public, only 22.8% of those described this engagement in trial publications—suggesting significant underreporting and highlighting how inconsistencies in reporting limit our ability to accurately assess variation in the scope, timing, and depth of engagement across the field.^60^

##### 3.6.5.3 Barriers to Meaningful and Sustained Engagement

Stakeholders across publications identified a consistent set of barriers that limit both the depth and sustainability of engagement in PCTs. Across roles and settings, engagement was frequently constrained by a combination of logistical, structural, and relational challenges. In a survey of PCORI Pilot Projects, investigators cited limited time (46%) and inadequate training (30%) as common obstacles to stakeholder involvement.^58^ Surveys with patients, caregivers, and clinicians echoed these concerns, emphasizing time pressures and unfamiliarity with engagement processes as recurring barriers.^59^, ^62^ Saunders et al. found that adolescent and parent stakeholders faced difficulties maintaining involvement over time due to life‐stage transitions and logistical challenges, compounded by unclear roles and power differentials in multi‐stakeholder settings.^63^ Additional publications highlighted that clinician‐perceived patient barriers (e.g., time, language, knowledge gaps)^64^ and physician reluctance to adopt research protocols that disrupted workflows^24^ further challenged efforts to establish meaningful, long‐term engagement.

## 4 DISCUSSION

This scoping review identified a substantial empirical ethics literature examining a wide variety of ethical issues encountered in PCTs (see Table 3).^65^ In addition, our review includes a preliminary synthesis of the five most prevalent themes that were identified in the literature: consent/disclosure; risk assessment; trust and transparency; burdens, barriers, and costs; and engagement.

Most publications included in this scoping review were conducted in the United States, with a notable proportion originating from Canada, followed by fewer publications from the UK, Australia, and New Zealand. This geographical concentration of PCT ethics research in the U.S. and Canada^66^ may reflect a higher prevalence of PCTs in these countries, a greater focus on ethical analysis within these research communities, the influence of targeted funding, the presence of particular research groups, or other reasons. Our review was not designed to systematically assess these factors, but they warrant consideration in interpreting the geographic distribution of the published literature we identified.

As PCTs expand globally, new ethical considerations will emerge, shaped by cultural factors and trust in healthcare systems. For example, Spaniards' high trust in their local physicians and healthcare system supports general notification over written consent, unlike in the U.S., while distrust in Kenya's healthcare system drives privacy concerns.^31^, ^40^, ^46^, ^67^ These differences caution against extrapolating U.S.‐based ethical insights to other settings. Expanding empirical ethics research to diverse cultural and healthcare settings through collaboration with local researchers can help to ensure findings are culturally relevant and perhaps support the development of appropriate ethical frameworks.

Just as in empirical bioethics research in general,^68^ this review reveals the predominant use of surveys (*n* = 39/82) as the primary research method in studies related to PCTs. Surveys are valuable for capturing broad perspectives and identifying widespread trends or common ethical concerns, particularly in the early stages of research when empirical data help frame the scope of ethical inquiry.^69^ However, the reliance on surveys—often involving the use of hypothetical scenarios rather than real‐world experiences—may limit their ability to uncover the nuanced, context‐specific dimensions of ethical issues. Qualitative methods such as interviews and focus groups may be better suited for such purposes.

Although hypothetical scenarios are useful for exploring general attitudes and ethical intuitions, they may not fully capture how various stakeholders—such as patients, clinicians, and policymakers—navigate the complexities in actual PCTs. This gap suggests an opportunity to explore whether and how these attitudes vary in real‐world contexts, particularly among those actually included in PCTs. Nesting empirical studies within ongoing PCTs could provide richer, more actionable insights by grounding stakeholder feedback in lived experiences.^70^

Discrete choice experiments (DCEs) can advance understanding of stakeholder preferences in PCTs by simulating real‐world trade‐offs and revealing how ethical and practical values are balanced.^71^ Although nearly half of the survey studies in this review (*n* = 19/39) used experimental designs, only one employed a DCE.^22^ In that study, patients and physicians evaluated hypothetical dialysis trials that varied in consent approach, patient burden, and physician autonomy; willingness to participate was most influenced by the consent strategy used. This illustrates how DCEs can help illuminate which trial design features matter most to stakeholders—an approach that could yield robust ethical insights if applied more widely in future empirical research. Nevertheless, DCEs are constrained by the limited number of variables that can be integrated into scenarios and tend to have a high respondent burden. In addition, specialized expertise is required to design and interpret them, which may restrict their broader application.

## 5 LIMITATIONS

Our findings should be considered in light of several limitations. First, our search strategy, which relied on existing compilations and keyword‐based searches, may have missed relevant materials due to database constraints and keyword limitations.

Second, the variability and evolving terminology used to describe PCTs pose challenges for identifying relevant studies and synthesizing findings across diverse contexts. Nevertheless, to overcome this issue, we curated our review to include related concepts, such as CER, ROMP, LHSs, and pragmatic CRTs, ensuring broader capture of relevant literature.

Third, as mentioned earlier, the predominance of studies conducted in the US and Canada limits the generalizability of our findings to other contexts. Additionally, our focus on English‐language publications likely skews results toward English‐speaking regions, limiting insights from non‐English sources and underrepresented contexts.

Finally, due to space constraints and the breadth of the literature identified, we focused our analysis on the five most frequently identified themes. While this approach allowed us to provide a meaningful preliminary synthesis of some ethical issues, it limited our ability to explore other important themes—such as justice, equity, and data governance—in greater depth. However, by identifying the ethical issues that have been most and least studied, this review provides a foundation for more targeted analyses of thematic gaps and their normative implications.

## 6 CONCLUSION

This review synthesizes empirical ethics literature on PCTs, identifying prevalent themes found in it including consent and disclosure; risk assessment; trust and transparency; burdens, barriers, and costs; and engagement. The findings reveal significant insights into these areas. For instance, alternatives to consent are being explored to balance ethical and practical considerations, while engagement strategies were associated with enhanced trust and trial relevance. However, empirical evidence on the outcomes of these strategies remains limited. Risk perceptions vary across stakeholders, often leading to inconsistencies in ethical and regulatory oversight, while logistical and financial burdens continue to pose challenges for PCT implementation. Transparency and clear communication have emerged as critical components in maintaining participant trust and ensuring ethical trial conduct.

Critical gaps remain, with research concentrated in Western contexts and reliant on surveys and hypothetical scenarios, limiting generalizability and real‐world insights. Addressing these gaps with geographically inclusive studies, innovative methods, and nested empirical work is important for developing appropriate ethical guidelines for PCTs. Taken together, these findings provide a foundation for future work that more critically examines the empirical ethics landscape of PCTs and addresses persistent gaps in attention, representation, and normative analysis.

## AUTHOR CONTRIBUTIONS

JS and SRM contributed to the study concept and design; KRM and SRM contributed to the data analysis; KRM wrote the first draft of the manuscript and prepared the figures; all authors contributed to critical revisions of the manuscript; all authors read and approved the final manuscript.

## FUNDING INFORMATION

This work was supported within the National Institutes of Health (NIH) Pragmatic Trials Collaboratory through cooperative agreement U24AT009676 from the National Center for Complementary and Integrative Health (NCCIH), the National Institute of Allergy and Infectious Diseases (NIAID), the National Cancer Institute (NCI), the National Institute on Aging (NIA), the National Heart, Lung, and Blood Institute (NHLBI), the National Institute of Nursing Research (NINR), the National Institute of Minority Health and Health Disparities (NIMHD), the National Institute of Arthritis and Musculoskeletal and Skin Diseases (NIAMS), the NIH Office of Behavioral and Social Sciences Research (OBSSR), and the NIH Office of Disease Prevention (ODP). This work was also supported by the NIH through the NIH HEAL Initiative under award number U24AT010961. The content is solely the responsibility of the authors and does not necessarily represent the official views of the NCCIH, NIAID, NCI, NIA, NHLBI, NINR, NIMHD, NIAMS, OBSSR, or ODP, or the NIH or its HEAL Initiative.

## CONFLICT OF INTEREST STATEMENT

Jeremy Sugarman is a consultant to Merck KGaA and Merck; he is a member of the Clinical Advisory Panel for Aspen Neurosciences.

## Supporting information


**Data S1.** Supporting Information.

## Data Availability

The data that supports the findings of this study are available in the supplementary material of this article.
