# Supplementary material for: Empirical research related to the ethics of pragmatic clinical trials: A scoping review
Source: Learn Health Syst. 2025 Oct 4;10(1):e70041. doi: 10.1002/lrh2.70041 (PMC12812493; doi:10.1002/lrh2.70041)
Supplement: Supplementary file 1 — Data S1. Supporting Information. [file LRH2-10-e70041-s001.docx]

**Appendices**

**Appendix A: Search Strategy - PubMed**

The table below displays the search strings used in the PubMed database.

| **PubMed Search Strings** | | | | | | |
| --- | --- | --- | --- | --- | --- | --- |
| **1** | "Pragmatic Clinical Trials as Topic/ethics"[Mesh] |  |  |  |  |  |
| **2** | "Comparative Effectiveness Research/ethics"[Mesh] |  |  |  |  |  |
| **3** | (Pragmatic Clinical Trials as Topic[Mesh] OR "Practical Clinical Trial*"[tiab] OR "Pragmatic Trial*"[tiab] OR "Pragmatic Clinical Trial*"[tiab]) | AND | (“Empirical Research”[Mesh] OR “Empirical Research”[tiab] OR “Empirical studies”[tiab] OR “Empirical literature”[tiab] OR “Survey”[tiab]) | AND | (“Ethics, Research”[Mesh] OR "Research Ethics"[tiab] OR "Ethics, Research"[tiab]) |  |
| **4** | ("Pragmatic Clinical Trials as Topic"[Mesh] OR "Practical Clinical Trial*"[tiab] OR "Pragmatic Trial*"[tiab] OR "Pragmatic Clinical Trial*"[tiab]) | AND | ("Ethics, Research"[Mesh] OR "Research Ethics"[tiab]) |  |  |  |
| **5** | ("Learning Health System"[Mesh] OR "Learning Health System*"[tiab] OR "Learning Healthcare System*"[tiab] OR "Learning Health Communit*"[tiab] OR “Embedded Trial*”[tiab]) | AND | (“Ethics, Research”[Mesh] OR "Research Ethics"[tiab] OR "Ethics, Research"[tiab]) |  |  |  |
| **6** | (Learning Health System[Mesh] OR "Learning Health System*"[tiab] OR "Learning Healthcare System*"[tiab] OR "Learning Health Communit*"[tiab] OR “Embedded Trial*”[tiab]) | AND | (“Empirical Research”[Mesh] OR “Empirical Research”[tiab] OR “Empirical studies”[tiab] OR “Empirical literature”[tiab] OR “Survey”[tiab]) |  |  |  |
| **7** | (“Comparative Effectiveness Research/ethics”[Mesh] OR “Comparative Effectiveness Research”[Mesh] OR “Comparative Effectiveness Research”[tiab] OR “Comparative Effectiveness Stud*”[tiab] OR “Comparative stud*”[tiab]) | AND | (“Empirical Research”[Mesh] OR “Empirical Research”[tiab] OR “Empirical studies”[tiab] OR “Empirical literature”[tiab] OR “Survey”[tiab]) |  |  |  |
| **8** | ("Comparative Effectiveness Research/ethics"[Mesh] OR "Comparative Effectiveness Research"[Mesh] OR "Comparative Effectiveness Research"[tiab] OR "Comparative Effectiveness Stud*"[tiab] OR "Comparative stud*"[tiab]) | AND | ("Ethics, Research"[Mesh] OR "Research Ethics"[tiab]) |  |  |  |
| **9** | ("learning health care"[tiab] OR "learning health care system"[tiab] OR "learning healthcare system"[tiab]) | AND | ("Ethics, Research"[Mesh] OR "Research Ethics"[tiab]) |  |  |  |
| **10** | ("Pragmatic Clinical Trials as Topic"[Mesh] OR "Practical Clinical Trial*"[tiab] OR "Pragmatic Trial*"[tiab] OR "Pragmatic Clinical Trial*"[tiab] OR "Pragmatic Randomized Trial" [tiab] OR "Pragmatic Cluster Randomized Trial" [tiab]) | AND | ("survey" [tiab] OR "perspective" [tiab] OR "qualitative" [tiab] OR "view" [tiab] OR "interview" [tiab] OR "perception*" [tiab] OR "attitude*" [tiab]) |  |  |  |
| **11** | ("Comparative Effectiveness Research/ethics"[Mesh] OR "Comparative Effectiveness Research"[Mesh] OR "Comparative Effectiveness Research"[tiab] OR "Comparative Effectiveness Stud*"[tiab] OR "Comparative Effectiveness Trial*" [tiab]) | AND | ("survey" [tiab] OR "perspective" [tiab] OR "qualitative" [tiab] OR "view" [tiab] OR "interview" [tiab] OR "perception*" [tiab] OR "attitude*" [tiab]) |  |  |  |
| **12** | ("Pragmatic Clinical Trials as Topic"[Mesh] OR "Practical Clinical Trial*"[tiab] OR "Pragmatic Trial*"[tiab] OR "Pragmatic Clinical Trial*"[tiab]) | AND | (“Ethics, Research”[Mesh]) |  |  |  |

**Appendix B: Search Strategy - Embase**

The table below displays the search strings used in the Embase database.

| **Embase Search Strings** | | | | | | |
| --- | --- | --- | --- | --- | --- | --- |
| **1** | ('ethics'/exp OR 'ethics' OR 'ethical':ab,ti,kw OR 'ethics':ab,ti,kw) | AND | ('learning health system'/exp OR 'learning health system' OR 'learning health care community':ab,ti,kw OR 'learning health care system':ab,ti,kw OR 'learning health community':ab,ti,kw OR 'learning healthcare system':ab,ti,kw OR 'learning health system':ab,ti,kw) |  |  |  |
| **2** | ('randomized controlled trial (topic)'/exp OR 'randomized controlled trial (topic)' OR 'practical clinical trial*':ab,ti,kw OR 'pragmatic trial*':ab,ti,kw OR 'pragmatic clinical trial*':ab,ti,kw OR 'pragmatic rct*':ab,ti,kw) | AND | ('ethics'/exp OR 'ethics' OR 'ethical':ab,ti,kw OR 'ethics':ab,ti,kw) | AND | ('survey*':ab,ti,kw OR 'perspective*':ab,ti,kw OR 'qualitative':ab,ti,kw OR 'view*':ab,ti,kw OR 'interview*':ab,ti,kw OR 'perception*':ab,ti,kw OR 'attitude*':ab,ti,kw) |  |
| **3** | ((comparative AND effectiveness AND topic) OR (comparative AND effectiveness:ab,ti,kw)) | AND | ('ethics'/exp OR ethics OR ethical:ab,ti,kw OR ethics:ab,ti,kw) | AND | (survey:ab,ti,kw OR perspective:ab,ti,kw OR qualitative:ab,ti,kw OR view:ab,ti,kw OR interview:ab,ti,kw OR perception*:ab,ti,kw OR attitude*:ab,ti,kw) |  |
| **4** | ('research ethics'/exp OR 'research ethics') | AND | ('randomized controlled trial (topic)'/exp OR 'randomized controlled trial (topic)' OR 'pragmatic trial'/exp OR 'pragmatic trial' OR 'learning health system'/exp OR 'learning health system' OR 'research on medical practice' OR 'phase 4 clinical trial'/exp OR 'phase 4 clinical trial') | AND | ('survey':ab,ti,kw OR 'perspective':ab,ti,kw OR 'qualitative':ab,ti,kw OR 'view':ab,ti,kw OR 'interview':ab,ti,kw OR 'perception*':ab,ti,kw OR 'attitude*':ab,ti,kw) |  |

**Appendix C: List of Included Publications**

| **#** | **Author Name(s)** | **Publication Date** | **Publication Title** |
| --- | --- | --- | --- |
| 1 | Penelope Abbott, Deborah Askew, Chelsea Watego, Wendy CY Hu, Letitia Campbell, Claudette Tyson, Robyn Walsh, Sylvia Hussey, Kerrie Doyle, Hasantha Gunasekera, Amanda Jane Leach, Tim Usherwood, Jessica Armstrong-Kearns, Jennifer Reath | 2021 | Randomised clinical trial research within Aboriginal and Torres Strait Islander primary health services: a qualitative study |
| 2 | Joseph Ali, Robert Califf, Jeremy Sugarman | 2016 | Anticipated Ethics and Regulatory Challenges in PCORnet: The National Patient-Centered Clinical Research Network |
| 3 | Juli M. Bollinger, Gail Geller, Kevin Weinfurt, Elizabeth May, Stephanie R.Morain, Debra J. H.Mathews, and Jeremy Sugarman | 2020 | Patients' Views About the Disclosure of Collateral Findings in Pragmatic Clinical Trials: a Focus Group Study |
| 4 | Howard Brody, Sharon A. Croisant, Jerome W. Crowder, and Jonathan P. Banda | 2015 | Ethical Issues in Patient-Centered Outcomes Research and Comparative Effectiveness Research: A Pilot Study of Community Dialogue |
| 5 | Chris Gale, Matthew J Hyde, and Neena Modi | 2017 | Research ethics committee decision-making in relation to an efficient neonatal trial |
| 6 | Shazia H Chaudhry, Jamie C Brehaut, Jeremy M Grimshaw, Charles Weijer, Robert Boruch, Allan Donner, Martin P Eccles, Andrew D McRae, Raphael Saginur, Zoe C Skea, Merrick Zwarenstein, and Monica Taljaard | 2013 | Challenges in the research ethics review of cluster randomized trials: International survey of investigators |
| 7 | Mildred K. Cho, David Magnus, Melissa Constantine, Sandra Soo-Jin Lee, Maureen Kelley, Stephanie Alessi, Diane Korngiebel, Cyan James, Ellen Kuwana, Thomas H. Gallagher, Douglas Diekema, Alexander M. Capron, Steven Joffe, and Benjamin S. Wilfond | 2015 | Attitudes Toward Risk and Informed Consent for Research on Medical Practices: A Cross-sectional Survey |
| 8 | Katherine R. Courtright, Scott D. Halpern, Steven Joffe, Susan S. Ellenberg, Jason Karlawish, Vanessa Madden, Nicole B. Gabler, Stephanie Szymanski, Kuldeep N. Yadav, and Laura M. Dember | 2017 | Willingness to participate in pragmatic dialysis trials: the importance of physician decisional autonomy and consent approach |
| 9 | Rafael Dal-Ré, Antonio J. Carcas, & Xavier Carné | 2017 | Who is willing to participate in low-risk pragmatic clinical trials without consent? |
| 10 | Rafael Dal-Ré, Antonio J. Carcas, Xavier Carné, and David Wendler | 2017 | Patients' beliefs regarding informed consent for low-risk pragmatic trials |
| 11 | Rafael Dal-Ré, Antonio J. Carcas, Xavier Carné, and David Wendler | 2017 | Public preferences on written informed consent for low-risk pragmatic clinical trials in Spain |
| 12 | Rafael Dal-Ré, Cristina Avendaño-Solà, Anthonius de Boer, Stephan K. James, Frits R. Rosendaal, Richard Stephens, and John P.A. Ioannidis | 2019 | A limited number of medicines pragmatic trials had potential for waived informed consent following the 2016 CIOMS ethical guidelines |
| 13 | Rhoma Dhamanaskar and Jon Merz | 2020 | High-impact RCTs without prospective informed consent: a systematic review |
| 14 | Neal W Dickert, Kristopher A Hendershot, Candace D Speight, and Alexandra E Fehr | 2016 | Patients' views of consent in clinical trials for acute myocardial infarction: impact of trial design |
| 15 | Neal W. Dickert, David Wendler, Chandan M. Devireddy, Sara F. Goldkind, Yi-An Ko, Candace D. Speight, and Scott Y.H. Kim | 2018 | Understanding Preferences Regarding Consent for Pragmatic Trials in Acute Care |
| 16 | Mark Duffett, Karen E. Burns, Michelle E. Kho, François Lauzier, Maureen O. Meade, Donald M. Arnold, Neill K.J. Adhikari, François Lamontagne, and Deborah J. Cook | 2011 | Consent in critical care trials: a survey of Canadian research ethics boards and critical care researchers |
| 17 | Lauren E. Ellis & Nancy E. Kass | 2017 | How are PCORI-funded researchers engaging patients in research and what are the ethical implications? |
| 18 | Dalia M. Feltman and John D. Lantos | 2016 | Neonatologists' opinions about the "foreseeable risks" in comparative effectiveness research: Results from an online survey |
| 19 | Laura P. Forsythe, Lauren E. Ellis, Lauren Edmundson, Raj Sabharwal, Alison Rein, Kristen Konopka, and Lori Frank | 2015 | Patient and Stakeholder Engagement in the PCORI Pilot Projects: Description and Lessons Learned |
| 20 | Laura P Forsythe, Lori B Frank, Thomas A Workman, Amanda Borsky, Tandrea Hilliard, Daniel Harwell & Lauren Fayish | 2017 | Health researcher views on comparative effectiveness research and research engagement |
| 21 | Laura P Forsythe, Lori B Frank, Thomas A Workman, Tandrea Hilliard, Daniel Harwell & Lauren Fayish | 2017 | Patient, caregiver and clinician views on engagement in comparative effectiveness research |
| 22 | Laura P Forsythe, Lori Frank, Kara Odom Walker, Ayodola Anise, Natalie Wegener, Harlan Weisman, Gail Hunt & Anne Beal | 2015 | Patient and clinician views on comparative effectiveness research and engagement in research |
| 23 | Laura P. Forsythe, Kristin L. Carman, Victoria Szydlowski, Lauren Fayish, Laurie Davidson, David H. Hickam, Courtney Hall, Geeta Bhat, Denese Neu, Lisa Stewart, Maggie Jalowsky, Naomi Aronson, and Chinenye Ursla Anyanwu | 2019 | Patient Engagement In Research: Early Findings From The Patient-Centered Outcomes Research Institute |
| 24 | Alan S. Gerber, Eric M. Patashnik, David Doherty, and Conor Dowling | 2010 | The Public Wants Information, Not Board Mandates, From Comparative Effectiveness Research |
| 25 | Nina Gobat, Christopher C Butler, Jill Mollison, Nicholas A Francis, Micaela Gal, Victoria Harris, Steve A R Webb, John-Paul Byrne, Angela Watkins, Prasanth Sukumar, Kerenza Hood, and Alistair Nichol | 2019 | What the public think about participation in medical research during an influenza pandemic: an international cross-sectional survey |
| 26 | Thomas Hills, Alex Semprini, and Richard Beasley | 2018 | Pragmatic randomised clinical trials using electronic health records: general practitioner views on a model of a priori consent |
| 27 | Anita Ho, Soodabeh Joolaee, Michael McDonald, Don Grant, Michel M. White, Holly Longstaff, and Eirikur Palsson | 2023 | Navigating Informed Consent Requirements and Expectations in Cluster Randomized Trials: Research Ethics Board Members' and Researchers' Views |
| 28 | Erin Holve, Marianne Hamilton Lopez, Lisa Scott, and Courtney Segal | 2012 | A tall order on a tight timeframe: stakeholder perspectives on comparative effectiveness research using electronic clinical data |
| 29 | Shona Kalkman, Ghislaine J. M. W. van Thiel, Diederick E. Grobbee, Anna-Katharina Meinecke, Mira G. P. Zuidgeest, Johannes J. M. van Delden | 2016 | Stakeholders' views on the ethical challenges of pragmatic trials investigating pharmaceutical drugs |
| 30 | Sherrie H. Kaplan, Adrijana Gombosev, Sheila Fireman, James Sabin, Lauren Heim, Lauren Shimelman, Rebecca Kaganov, Kathryn E. Osann, Thomas Tjoa & Susan S. Huang | 2016 | The patient's perspective on the need for informed consent for minimal risk studies: Development of a survey-based measure |
| 31 | Nancy E. Kass, Ruth R. Faden, Stephanie R. Morain, and Kristina Hallez | 2020 | Are Shorter Informed Consent Discussions, Compared with Longer Written Forms, Acceptable to Patients for Low-Risk Comparative Studies? |
| 32 | Nancy E. Kass, Ruth R. Faden, Stephanie R. Morain, Kristina Hallez, Rebecca A. Stametz, Amanda R. Milo, and Deserae Clarke | 2022 | Streamlined versus traditional consent for low-risk comparative effectiveness trials: a randomized experimental study to measure patients' and public attitudes |
| 33 | Nancy Kass, Ruth Faden, Rachel E. Fabi, Stephanie Morain, Kristina Hallez, Danielle Whicher, Sean Tunis, Rachael Moloney, Donna Messner & James Pitcavage | 2016 | Alternative consent models for comparative effectiveness studies: Views of patients from two institutions |
| 34 | Maureen Kelley, Cyan James, Stephanie Alessi, Diane Korngiebel, Isabelle Wijangco, Emily Rosenthal, Steven Joffe, Mildred K. Cho, Benjamin Wilfond, and Sandra Soo-Jin Lee | 2015 | Patient Perspectives on the Learning Health System: the Importance of Trust and Shared Decision Making |
| 35 | Julia Kohn, Zoe Unger, Jennifer Dolatshahi, Hannah Simons & Alison Rein | 2017 | Attitudes toward comparative effectiveness research and patient engagement among reproductive health clinicians |
| 36 | Stephanie A Kraft, Melissa Constantine, David Magnus, Kathryn M. Porter, Sandra Soo-Jin Lee, Michael Green, Nancy E Kass, Benjamin S. Wilfond, and Mildred K Cho | 2017 | A randomized study of multimedia informational aids for research on medical practices: implications for informed consent |
| 37 | Stephanie Alessi Kraft, Mildred K. Cho, Melissa Constantine, Sandra Soo-Jin Lee, Maureen Kelley, Diane Korngiebel, Cyan James, Ellen Kuwana, Adrienne Meyer, Kathryn Porter, Douglas Diekema, Alexander M. Capron, Radica Alicic, Benjamin S Wilfond, and David Magnus | 2016 | A Comparison of Institutional Review Board Professionals' and Patient Views on Consent for Research on Medical Practices |
| 38 | Stephanie A. Kraft, Kathryn M. Porter, Diane M. Korngiebel, Cyan James, Melissa Constantine, Maureen Kelley, Alexander M. Capron, Douglas Diekema, Sandra Soo-Jin Lee, Mildred K. Cho, David Magnus, Benjamin S. Wilfond | 2017 | Research on Medical Practices: Why Patients Consider Participating and the Investigational Misconception |
| 39 | Ashley Kraybill, Laura M. Dember, Steven Joffe, Jason Karlawish, Susan S. Ellenberg, Vanessa Madden, and Scott D. Halpern | 2016 | Patient and Physician Views about Protocolized Dialysis Treatment in Randomized Trials and Clinical Care |
| 40 | Ernest Law, Rachel Harrington, G Caleb Alexander, Soumi Saha, Elisabeth Oehrlein, and Eleanor M Perfetto | 2018 | Increasing uptake of comparative effectiveness and patient-centered outcomes research among stakeholders: insights from conference discussion |
| 41 | Sandra Soo-Jin Lee, Maureen Kelley, Mildred K. Cho, Stephanie Alessi Kraft, Cyan James, Melissa Constantine, Adrienne N. Meyer, Douglas Diekema, Alexander M. Capron, Benjamin S. Wilfond & David Magnus | 2016 | Adrift in the gray zone: IRB perspectives on research in the learning health system |
| 42 | Eric J. Lenze, Alex Ramsey, Patrick J. Brown, Charles F. Reynolds III, Benoit H. Mulsant, Helen Lavretsky, and Steven P. Roose | 2016 | Older Adults’ Perspectives on Clinical Research: A Focus Group and Survey Study |
| 43 | Sarah J. Lowry, Elizabeth T. Loggers, Erin J.A. Bowles, and Edward H. Wagner | 2012 | Evidence Gaps in Advanced Cancer Care: Community-Based Clinicians’ Perspectives and Priorities for Comparative Effectiveness Research |
| 44 | Kathleen M Mazor, Allison Richards, Mia Gallagher, David E Arterburn, Marsha A  Raebel, W Benjamin Nowell, Jeffrey R Curtis, Andrea R Paolino & Sengwee Toh | 2017 | Stakeholders’ views on data sharing in  multicenter studies |
| 45 | Daniel Mbuthia, Sassy Molyneux, Maureen Njue, Salim Mwalukore, and Vicki Marsh | 2019 | Kenyan health stakeholder views on individual consent, general notification and governance processes for the re-use of hospital inpatient data to support learning on healthcare systems |
| 46 | Jenny McLeish, Fiona Alderdice, Helen Robberts, Christina Cole, Jon Dorling, and Chris Gale | 2021 | Challenges of a simplified opt-out consent process in a neonatal randomised controlled trial: qualitative study of parents' and health professionals' views and experiences |
| 47 | Stuart McLennan | 2019 | The ethical oversight of learning health care activities in Switzerland: a qualitative study |
| 48 | Andrew D McRae, Carol Bennett, Judith Belle Brown, Charles Weijer, Robert Boruch, Jamie Brehaut, Shazia Chaudhry, Allan Donner, Martin Eccles, Jeremy Grimshaw, Merrick Zwarenstein, and Monica Taljaard | 2013 | Researchers' perceptions of ethical challenges in cluster randomized trials: a qualitative analysis |
| 49 | Rahul Mhaskar, Barry B. Bercu, and Benjamin Djulbegovic | 2013 | At What Level of Collective Equipoise Does a Randomized Clinical Trial Become Ethical for the Members of Institutional Review Board/Ethical Committees? |
| 50 | David Gibbes Miller, Scott Y. H. Kim, Xiaobai Li, Neal W. Dickert, James Flory, Carlisle P. Runge, and Clare Relton | 2018 | Ethical Acceptability of Postrandomization Consent in Pragmatic Clinical Trials |
| 51 | Stephanie R. Morain and Nancy E. Kass | 2016 | Ethics Issues Arising in the Transition to Learning Health Care Systems: Results from Interviews with Leaders from 25 Health Systems |
| 52 | Stephanie R. Morain, Nancy E. Kass, and Ruth R. Faden | 2021 | What Factors Predict Willingness to Join Low-Risk Pragmatic Clinical Trials? |
| 53 | Stephanie R. Morain, Ellen Tambor, Rachael Moloney, Nancy E. Kass, Sean Tunis, Kristina Hallez, and Ruth R. Faden | 2018 | Stakeholder perspectives regarding alternate approaches to informed consent for comparative effectiveness research |
| 54 | Stephanie R. Morain, Debra J.H. Matthews, Kevin Weinfurt, Elizabeth May, Juli M. Bollinger, Gail Geller, and Jeremy Sugarman | 2021 | Stakeholder perspectives regarding pragmatic clinical trial collateral findings |
| 55 | Stephanie R. Morain, Juli Bollinger, Kevin Weinfurt, and Jeremy Sugarman | 2024 | Stakeholder perspectives on data sharing from pragmatic clinical trials: Unanticipated challenges for meeting emerging requirements |
| 56 | Rahul N. Nayak, David Wendler, Franklin G. Miller, and Scott Y.H. Kim | 2015 | Pragmatic Randomized Trials Without Standard Informed Consent?: A National Survey |
| 57 | Rahul K. Nayak and David Wendler | 2017 | Is it important to disclose how treatments are selected in clinical research and clinical care? |
| 58 | Stuart G. Nicholls, Kelly Carroll, Hayden P. Nix, Fan Li, Specer Phillips Hey, Susan L. Mitchell, Charles Weijer, and Monica Taljaard | 2022 | Ethical considerations within pragmatic randomized controlled trials in dementia: Results from a literature survey |
| 59 | Stuart G. Nicholls, Kelly Carroll, Cory E. Goldstein, Jamie C. Brehaut, Charles Weijer, Merrick Zwarenstein, Stephanie Dixon, Jeremy M. Grimshaw, Amit X. Garg, and Monica Taljaard | 2021 | Patient Partner Perspectives Regarding Ethically and Clinically Important Aspects of Trial Design in Pragmatic Cluster Randomized Trials for Hemodialysis |
| 60 | Stuart G. Nicholls, Kelly Carroll, Charles Weijer, Cory E. Goldstein, Jamie Brehaut, Manish M. Sood, Ahmed Al-Jaishi, Erika Basile, Jeremy M. Grimshaw, Amit X. Garg, and Monica Taljaard | 2020 | Ethical Issues in the Design and Conduct of Pragmatic Cluster Randomized Trials in Hemodialysis Care: An Interview Study With Key Stakeholders |
| 61 | Stuart G. Nicholls, Kelly Carroll, Merrick Zwarenstein, Jamie C. Brehaut, Charles Weijer, Spencer P. Hey, Cory E. Goldstein, Ian D. Graham, Jeremy M. Grimshaw, Joanne E. McKenzie, Dean A. Fergusson, Monica Taljaard | 2019 | The ethical challenges raised in the design and conduct of pragmatic trials: an interview study with key stakeholders |
| 62 | Stuart G. Nicholls, Ahmed A. Al-Jaishi, Harrison Niznick, Kelly Carroll, Mohamad Tarek Madani2 Katherine D. Peak3 Leen Madani2 Pascale Nevins1 Lionel Adisso4 Fan Li5 Charles Weijer6 Susan L. Mitchell7 Vivian Welch8 Ana R. Quiñones3 Monica Taljaard9 | 2023 | Health equity considerations in pragmatic trials in Alzheimer’s and dementia disease: Results from a methodological review |
| 63 | Shelley Potter, Nicola Mills, Simon J. Cawthorn, Jenny Donovan, and Jane M. Blazeby | 2014 | Time to be BRAVE: is educating surgeons the key to unlocking the potential of randomised clinical trials in surgery? A qualitative study |
| 64 | Emily J. Rozema, Beth Creekmur, Visanee V. Musigdilok, Jennifer Steltz, Michael K. Gould, and Christopher G. Slatore | 2022 | Patient responses to passive enrollment into a large, pragmatic clinical trial: A qualitative content analysis |
| 65 | Lisa Caputo Sandy, Thomas J. Glorioso, Kevin Weinfurt, Jeremy Sugarman, Pamela N. Peterson, Russell E. Glasgow, and P. Michael Ho | 2021 | Leave me out: Patients' characteristics and reasons for opting out of a pragmatic clinical trial involving medication adherence |
| 66 | Tully Saunders, Thomas I. Mackie, Supriya Shah, Holly Gooding, Sarah D. de Ferranti, & Laurel K. Leslie | 2016 | Young adult and parent stakeholder perspectives on participation in patient-centered comparative effectiveness research |
| 67 | Alex Semprini, Thomas Hills, Irene Braithwaite, Mark Weatherall, and Richard Beasley | 2017 | A priori consent within pragmatic randomised controlled trials: a web-based survey of statin use in primary care |
| 68 | Gina M. Sgro, Maureen Maurer, Beth Nguyen, and Joanna E. Siegel | 2023 | Return of aggregate results to study participants: Facilitators, barriers, and recommendations |
| 69 | Tanya J. Symons, Nicola Straiton, Rosie Gagnon, Roberta Littleford, Anita J. Campbell, Asha C. Bowen, Adam G. Stewart, Steven Y.C. Tong, and Joshua S. Davis | 2022 | Consumer perspectives on simplified, layered consent for a low risk, but complex pragmatic trial |
| 70 | Monica Taljaard, Jamie C Brehaut, Charles Weijer, Robert Boruch, Allan Donner, Martin P Eccles, Andrew D McRae, Raphael Saginur, Merrick Zwarenstein, and Jeremy M Grimshaw | 2014 | Variability in research ethics review of cluster randomized trials: a scenario-based survey in three countries |
| 71 | Monica Taljaard, Shazia H Chaudhry, Jamie C Brehaut, Charles Weijer, Robert Boruch, Allan Donner, Martin P Eccles, Andrew D McRae, Raphael Saginur, Merrick Zwarenstein, and Jeremy M Grimshaw | 2014 | Survey of consent practices in cluster randomized trials: Improvements are needed in ethical conduct and reporting |
| 72 | Monica Taljaard, Cory E Goldstein, Bruno Giraudeau, Stuart G Nicholls, Kelly Carroll, Spencer Phillips Hey, Jamie C Brehaut, Vipul Jairath, Alex John London, Sandra M Eldridge, Jeremy M Grimshaw, Dean A Fergusson, and Charles Weijer | 2020 | Cluster over individual randomization: are study design choices appropriately justified? Review of a random sample of trials |
| 73 | Rachel Topazian, Juli Bollinger, Kevin P Weinfurt, Rachel Dvoskin, Debra Mathews, Kathleen Brelsford, Matthew DeCamp & Jeremy Sugarman | 2016 | Physicians' perspectives regarding pragmatic clinical trials |
| 74 | Shelley Vanderhout, Pascale Nevins, Stuart G. Nicholls, Colin Macarthur, Jamie C. Brehaut, Beth K. Potter, Kate Gillies, Beatriz Goulao, Maureen Smith, Alicia Hilderley, Kelly Carroll, Anne Spinewine, Charles Weijer, Dean A. Fergusson, and Monica Taljaard | 2023 | Patient and public involvement in pragmatic trials: online survey of corresponding authors of published trials |
| 75 | Kevin P. Weinfurt, Juli M. Bollinger, Kathleen M. Brelsford, Travis J. Crayton, Rachel J. Topazian, Nancy E. Kass, Laura M. Beskow & Jeremy Sugarman | 2016 | Patients' views concerning research on medical practices: Implications for consent |
| 76 | Kevin P Weinfurt, Li Lin, and Jeremy Sugarman | 2019 | Public views regarding the responsibility of patients, clinicians, and institutions to participate in research in the United States |
| 77 | Kevin P. Weinfurt, Juli M. Bollinger, Kathleen M. Brelsford, Martina Bresciani, Zachary Lampron, Li Lin, Rachel J. Topazian, and Jeremy Sugarman | 2017 | Comparison of Approaches for Notification and Authorization in Pragmatic Clinical Research Evaluating Commonly Used Medical Practices |
| 78 | Kevin P. Weinfurt, Juli M. Bollinger, Elizabeth May, Gail Geller, Debra J. H. Mathews, Stephanie R. Morain, Lorrie Schmid, Diane L. Bloom, and Jeremy Sugarman | 2021 | Patients' Reactions to Letters Communicating Collateral Findings of Pragmatic Clinical Trials: a National Web-Based Survey |
| 79 | Charlene R Weir, Jorie Butler, Iona Thraen, Patricia A Woods, John Hermos, Ryan Ferguson, Theresa Gleason, Robyn Barrus and Louis Fiore | 2014 | Veterans Healthcare Administration providers' attitudes and perceptions regarding pragmatic trials embedded at the point of care |
| 80 | Danielle Whicher, Nancy Kass, and Ruth Faden | 2015 | Stakeholders' Views of Alternatives to Prospective Informed Consent for Minimal-Risk Pragmatic Comparative Effectiveness Trials |
| 81 | Danielle Whicher, Nancy Kass, Yashar Saghai, Ruth Faden, Sean Tunis, and Peter Pronovost | 2015 | The Views of Quality Improvement Professionals and Comparative Effectiveness Researchers on Ethics, IRBs, and Oversight |
| 82 | Jennifer Zhe Zhang, Stuart G Nicholls, Kelly Carroll, Hayden Peter Nix, Cory E Goldstein, Spencer Phillips Hey, Jamie C Brehaut, Paul C McLean, Charles Weijer, Dean A Fergusson, and Monica Taljaard | 2023 | Informed consent in pragmatic trials: results from a survey of trials published 2014-2019 |
